# An international legal framework to address antimicrobial resistance

**DOI:** 10.2471/BLT.15.152710

**Published:** 2015-02-01

**Authors:** Steven J Hoffman, Kevin Outterson, John-Arne Røttingen, Otto Cars, Charles Clift, Zain Rizvi, Fiona Rotberg, Göran Tomson, Anna Zorzet

**Affiliations:** a Global Strategy Lab, Faculty of Law, University of Ottawa, 57 Louis Pasteur Street, Ottawa, Ontario, K1N 6N5, Canada.; b School of Law, Boston University, Boston, United States of America (USA).; c Division of Infectious Disease Control, Norwegian Institute of Public Health, Oslo, Norway.; d ReAct – Action on Antibiotic Resistance, Uppsala University, Uppsala, Sweden.; e Centre on Global Health Security, Chatham House, London, England.; f Yale Law School, Yale University, New Haven, USA.; g Dag Hammarskjöld Foundation, Uppsala, Sweden.; h Department of Learning, Informatics, Management and Ethics, Karolinska Institute, Stockholm, Sweden.

Antimicrobial resistance is recognized as a grave threat to global health.^1^^,^^2^ It already causes an estimated 700 000 deaths annually and – without effective action – is predicted to cause 10 million deaths annually by 2050.^3^ The World Health Organization (WHO) has prepared a draft *Global action plan on antimicrobial resistance* that will be discussed at this year’s World Health Assembly.^4^ However, more is required if the world is to grapple effectively with this huge and complex problem. Global collective action is required in three areas: (i) access, to ensure that the prevention tools, diagnostics and therapies needed to reduce the infectious disease burden are available and affordable to everyone, everywhere; (ii) conservation, to reduce the need for antimicrobials and ensure their responsible use through prevention efforts, infection control, surveillance and appropriate prescriptions; and (iii) innovation, to develop the next generation of antimicrobials, vaccines, diagnostics and infection control technologies.

The problem of antimicrobial resistance requires that all three areas be tackled simultaneously. Without conservation and innovation, universal access will simply drive resistance and deplete existing stocks of effective antimicrobials. Conservation, if pursued alone, will constrict the market for antimicrobials, restrict investment and innovation in the field and hinder access.^5^ Innovation without conservation will waste new drugs and diminish the value of investments. Innovation without better access is inequitable. Like the legs of a tripod, each area needs the support of the other two. However, solving the issues of access, conservation and innovation simultaneously will require new coordination and financing mechanisms, some of which must be organized globally.

To avert millions of deaths caused by treatable infections, access to antimicrobials should be scaled-up for the many people worldwide who cannot obtain or afford such drugs. Access could be facilitated by equitable pricing or licensing models, but external resources will be required to subsidize access for the world’s poorest people. Such subsidies create common benefit, by reducing disease transmission and preventing reservoirs of resistant pathogens created by inconsistent use.

Conservation activities should continue to be directed by national and local governments but global standards are needed for surveillance, infection control, health-worker training, sales promotion, direct-to-consumer advertising and safeguards against incentives for overuse.^6^

Although public innovation funding will realistically continue to flow mostly from national budgets, stronger coordination is needed among key research funders and commercial investors in innovation. Some funding and rewards should also be pooled globally. To avoid incentives for overuse, rewards will need to be delinked, entirely or partially, from volume of sales.^7^^,^^8^ The financial contributions from countries should be differentiated according to their means.

Given these global coordination issues, there is a clear role for a binding international legal framework to encompass the issues of access, conservation and innovation. When paired with strong implementation mechanisms,^9^ international law represents the strongest possible way in which countries can commit themselves to act.^10^ Where and how should this be done? While a small number of high-income countries could make progress on innovation,^11^ long-term success on conservation and access requires nearly universal participation. Several options could be explored but two seem particularly salient and should be pursued in parallel. One is the development of a new WHO regulation, under Article 21 of WHO’s Constitution, that is akin to, but separate from, the International Health Regulations.^12^ Any Article 21 regulation is automatically binding on all WHO’s Member States – unless a Member State opts out. The second option is the development of a new international treaty negotiated under the auspices of the United Nations General Assembly.

Our future health depends on forming an international legal framework that resolves – or at least substantially reduces – the problem of antimicrobial resistance.
